# Publisher Correction: Heme enables proper positioning of Drosha and DGCR8 on primary microRNAs

**DOI:** 10.1038/s41467-018-06426-4

**Published:** 2018-09-18

**Authors:** Alexander C. Partin, Tri D. Ngo, Emily Herrell, Byung-Cheon Jeong, Gary Hon, Yunsun Nam

**Affiliations:** 1 Cecil H. and Ida Green Center for Reproductive Biology Sciences and Division of Basic Reproductive Biology Research, Department of Obstetrics and Gynecology, University of Texas Southwestern Medical Center, Dallas, TX 75390 USA; 2 Department of Biophysics, University of Texas Southwestern Medical Center, Dallas, TX 75390 USA

Correction to: *Nature Communications*; 10.1038/s41467-017-01713-y, published online 23 Nov 2017.

The original version of this Article contained an error in Fig. 1. In panel d, the model on the right of the panel was incorrectly labeled ‘+Heme’, and should have read ‘− Heme’. This has now been corrected in both the PDF and HTML versions of the Article.

