# Supplementary material for: Heme enables proper positioning of Drosha and DGCR8 on primary microRNAs
Source: Nat Commun. 2017 Nov 23;8:1737. doi: 10.1038/s41467-017-01713-y (PMC5700927; doi:10.1038/s41467-017-01713-y)
Supplement: Supplementary file 1 — Supplementary Information [file 41467_2017_1713_MOESM1_ESM.pdf]

Supplementary Figure 1

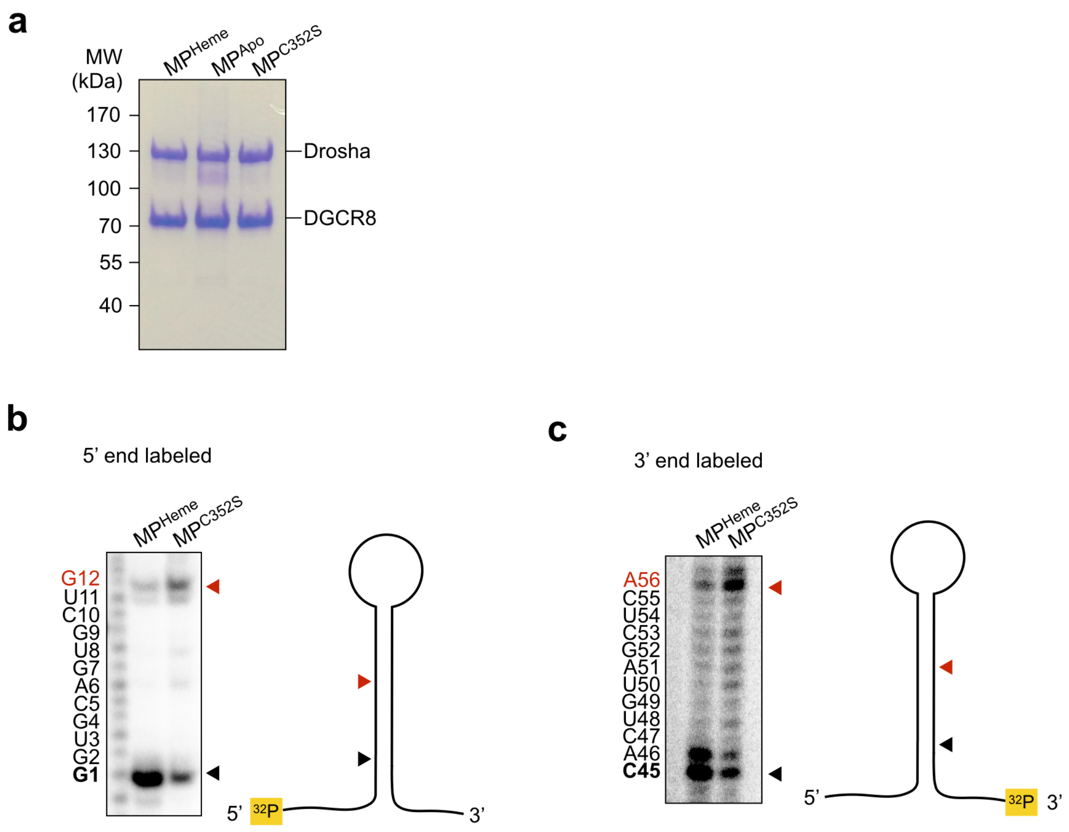

Supplementary Figure 1. Heme binding reverses the orientation of Microprocessor on pri-miRs.

(**a**) SDS-PAGE showing MP^{Heme}, MP^{Apo}, and MP^{C352S} used in this study.
(**b-c**) Representative images of sequencing polyacrylamide/urea gels used to identify Microprocessor cleavage sites, using (**b**) 5’ and (**c**) 3’ end-labeled pri-miR-143. RNAse T1 (not shown) and alkaline hydrolysis ladders were used to map the cut sites for MP^{Heme} and MP^{C352S}. Cut sites associated with MP^{Heme} and MP^{C352S} are shown in black and red, respectively.

Supplementary Figure 2

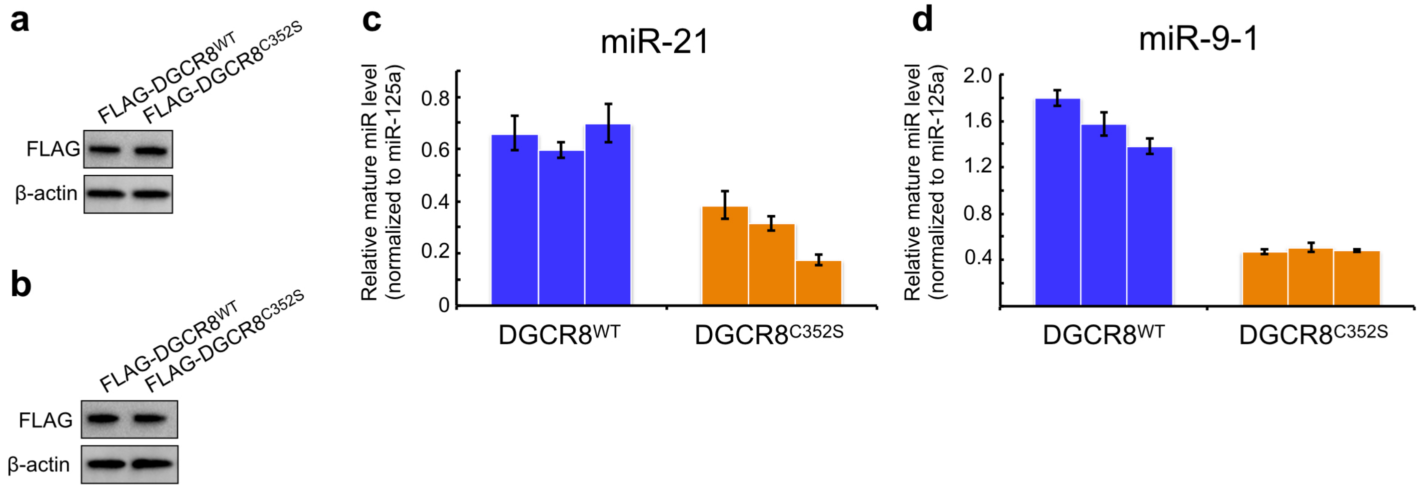

Supplementary Figure 2. Heme dependence varies among miRs.

(a-b) Western blots for transfections described in **Fig. 2b-g**, for (**a**) pri-21 and (**b**) pri-9-1 experiments.
(**c-d**) Taqman quantitative PCR assay results from transfections in **Fig. 2b-c**, for miR-21 **(c)** and miR-9-1 **(d)**. Mature miR levels were normalized to miR-125a levels using the comparative C_T method. Values represented are mean ± standard deviation from a total of 4 technical replicates, and each bar represents an independent transfection (biological replicate).

Supplementary Figure 3

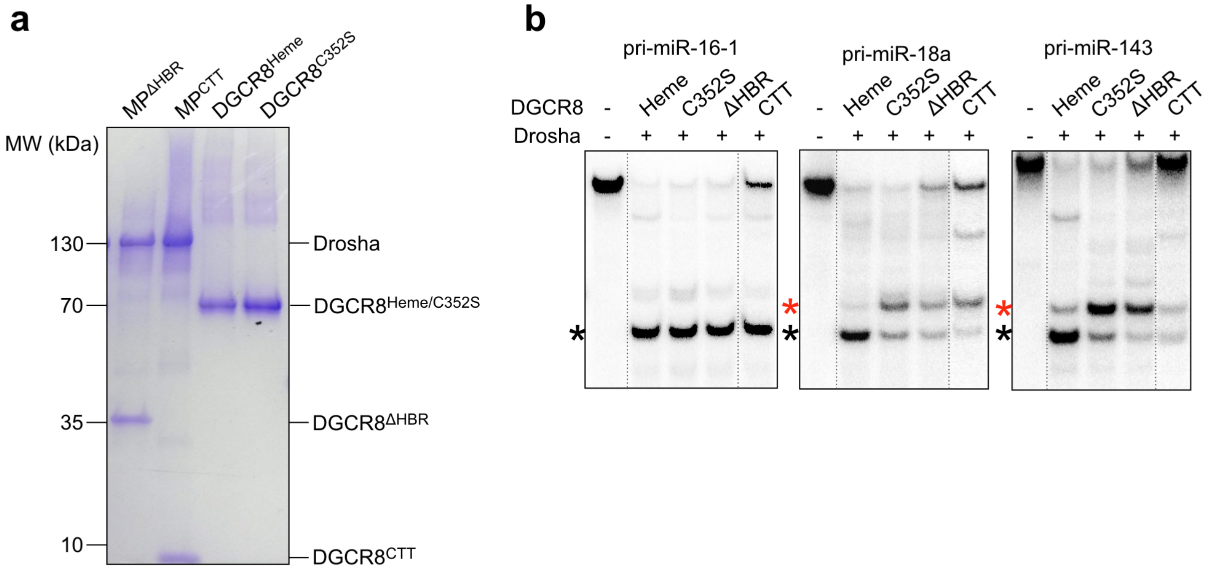

Supplementary Figure 3. Heme enables DGCR8 to guide Drosha to the correct junction.

(**a**) SDS-PAGE of recombinant MP$^{\Delta HBR}$, MP^{CTT}, DGCR8^{Heme} and DGCR8^{C352S} stained with Coomassie Blue. (**b**) Additional 5' end-labeled in vitro processing assays comparing DGCR8 titrations (see **Fig. 3d**). MP^{Heme} (130 nM), MP^{C352S} (130 nM), MP$^{\Delta HBR}$ (260 nM), and MP^{CTT} (1.04 μM) reactions are shown.

Supplementary Figure 4

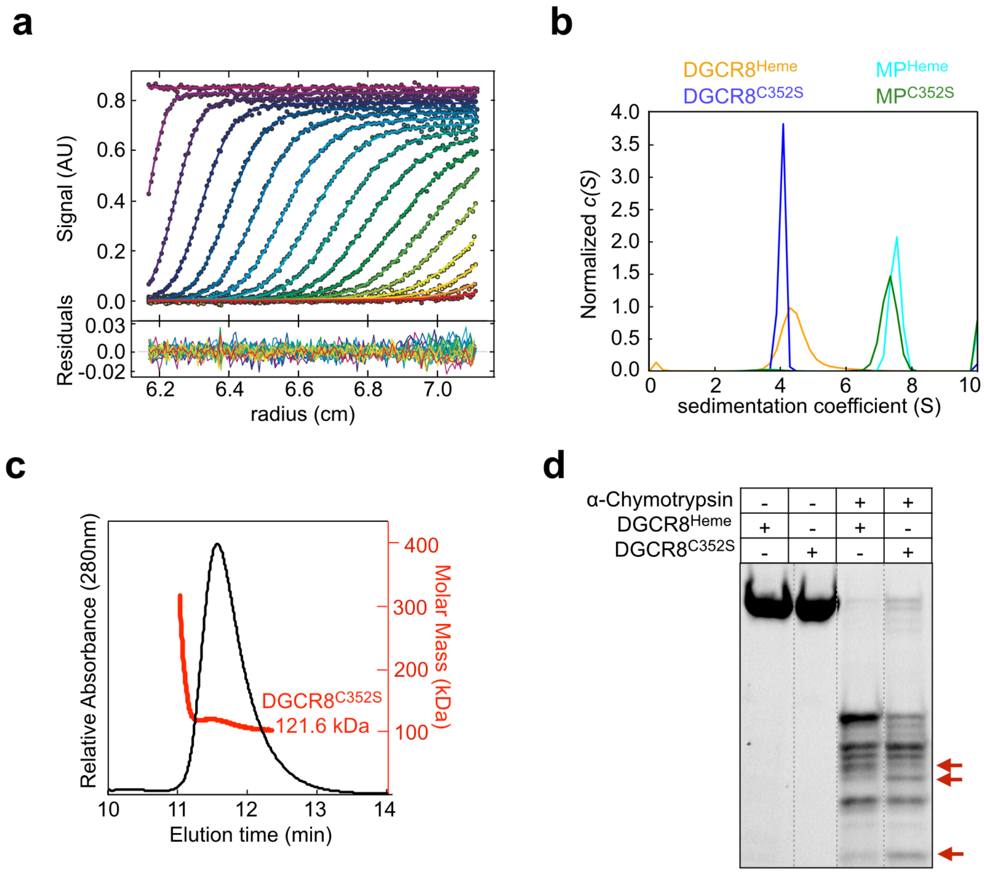

Supplementary Figure 4. Heme induces a conformational change rather than promoting dimerization of DGCR8.

(a) Representative radial scans for MP^{Heme}, with residuals shown below for fit quality. Movement of the boundary region is indicated by color spectrum, with earliest time points in violet and later time points in red. Data curves were created using GUSSI.
(b) Graph showing c(S) analysis and sedimentation coefficients for $DGCR8^{Heme}$, $DGCR8^{C352S}$, MP^{Heme} and MP^{C352S}. Curves were created using GUSSI.
(c) SEC-MALS analysis reports a molecular mass of 121.6 kDa for the heme-binding mutant $DGCR8^{C352S}$, indicating stable dimeric state (calculated mass of dimer: 120.9 kDa)
(d) Stain-free SDS-PAGE showing proteolytic digestion of $DGCR8^{Heme}$ and $DGCR8^{C352S}$. Differential degradation pattern (red arrows) suggests an altered conformation.

Supplementary Figure 5

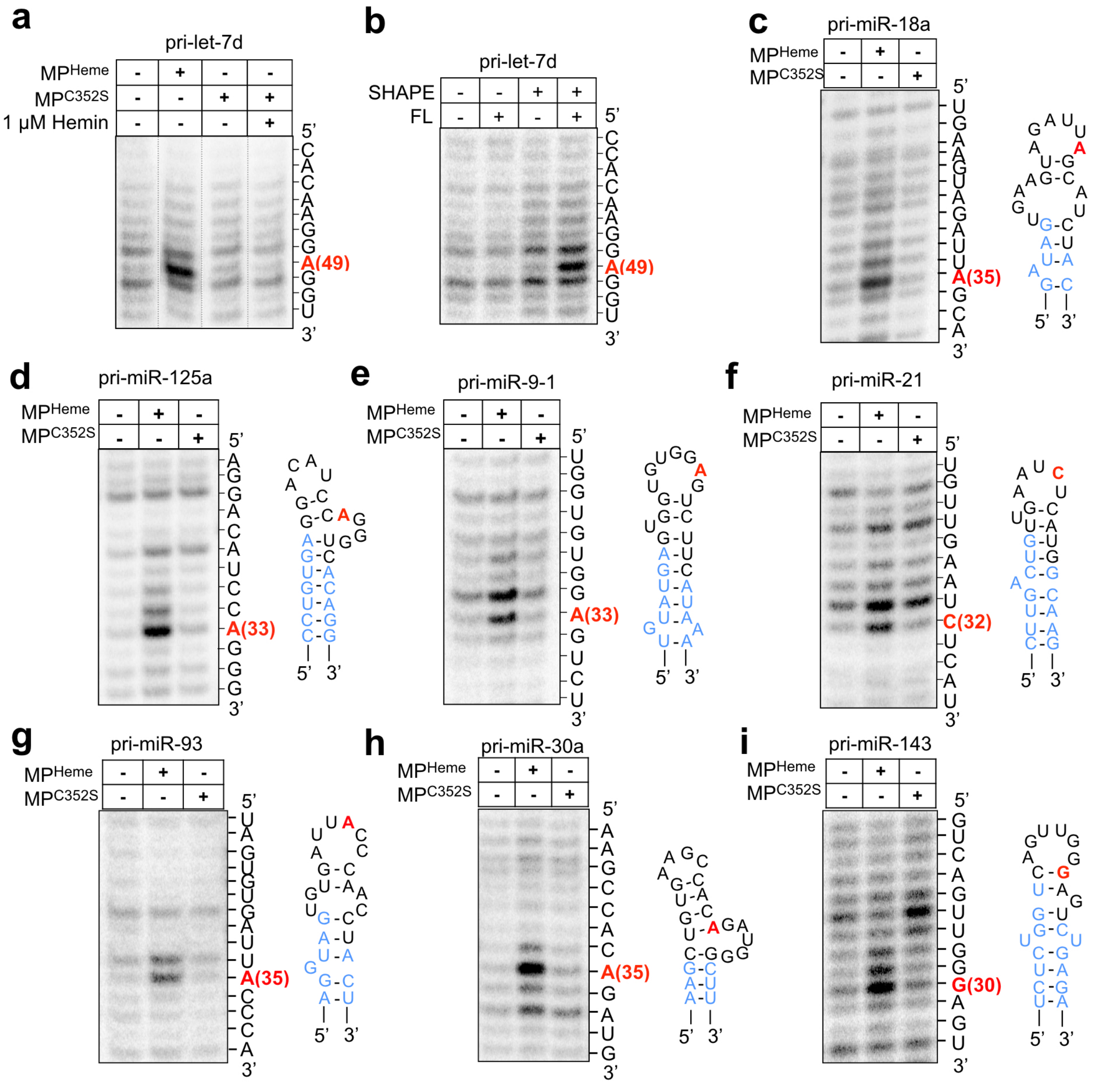

Supplementary Figure 5. Heme binding allows DGCR8 to recognize the terminal loop structure.

(a) SHAPE analysis of pri-let-7d, indicating that addition of 1 µM hemin chloride to Microprocessor incapable of binding heme does not restore hyperreactivity observed with heme-saturated Microprocessor.
(b) SHAPE analysis of pri-let-7d, with full-length Drosha and full-length DGCR8 purified from 293T cells. Control reactions containing no SHAPE reagent (BzCN) are shown (lanes 1-2).
(c-i) SHAPE analysis of pri-miRs, **(c)** -18a, **(d)** -125a, **(e)** 9-1, **(f)** -21, **(g)** -93, **(h)** -30a, and **(i)** -143 with secondary structure diagrams shown to the right. SHAPE hotspot associated with wild type MP^{Heme} is marked in red. Mature sequence is shown in light blue. All secondary structure diagrams were designed using mfold.

Supplementary Figure 6

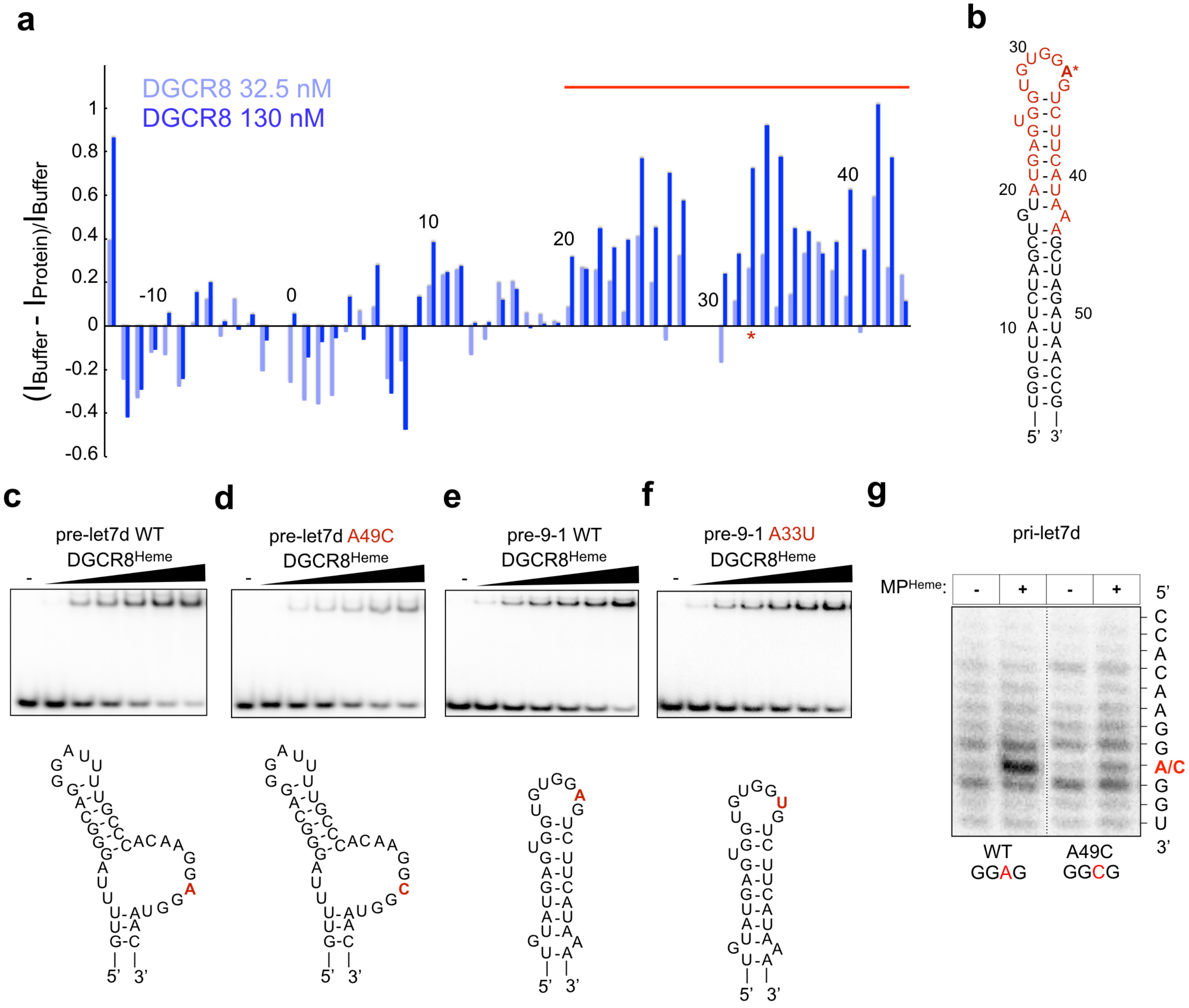

Supplementary Figure 6. Heme binding allows DGCR8 to recognize the terminal loop structure.

(**a**) Quantitation of hydroxyl radical footprinting experiments for pri-miR-9-1 and DGCR8^{Heme}, at a final protein concentration of 32.5 nM (light blue) and 130 nM (dark blue). From left to right, the bars represent nucleotides in the 5' to 3' direction, numbered as in (**b**). The A33 nucleotide that is hyperreactive in SHAPE assays in indicated with an asterisk.
(**b**) Secondary structure diagram of pri-miR-9-1 illustrating locations of protected sites from hydroxyl radical footprinting experiments, with red bases corresponding to the line shown above bars in (**a**). A33 is indicated with an asterisk.
(**c-d)** EMSAs comparing binding affinities of DGCR8^{Heme} for **(c)** wild-type pre-let-7d and **(d)** a pre-let-7d point mutant in which the "hotspot" adenine has been mutated to a cytosine. Protein concentrations were (left to right): 33.3 nM, 66.7 nM, 100 nM, 133.3 nM, 166.7 nM, and 200 nM.
(e-f) EMSAs comparing binding affinities of DGCR8^{Heme} for **(e)** wild-type pre-miR-9-1 and **(f)** a pre-miR-9-1 point mutant in which the "hotspot" adenine has been mutated to a uracil. Protein concentrations were (left to right): 33.3 nM, 66.7 nM, 100 nM, 133.3 nM, 166.7 nM, and 200 nM.
(**g**) SHAPE analysis of pri-let-7d used in (**c-d**).
All secondary structure diagrams were designed using mfold.
